# A UAV Maneuver Decision-Making Algorithm for Autonomous Airdrop Based on Deep Reinforcement Learning

**DOI:** 10.3390/s21062233

**Published:** 2021-03-23

**Authors:** Ke Li, Kun Zhang, Zhenchong Zhang, Zekun Liu, Shuai Hua, Jianliang He

**Affiliations:** 1 School of Electronics and Information, Northwestern Polytechnical University, Xi’an 710072, China; keli_iat@mail.nwpu.edu.cn (K.L.); connor_zhang@mail.nwpu.edu.cn (Z.Z.); lzk_nwpu@mail.nwpu.edu.cn (Z.L.); huas@mail.nwpu.edu.cn (S.H.); 2 Science and Technology on Electro-Optic Control Laboratory, Luoyang 471009, China; hejianlian_457@163.com

**Keywords:** UAV, maneuver decision-making, autonomous airdrop, deep reinforcement learning, prioritized experience replay

## Abstract

How to operate an unmanned aerial vehicle (UAV) safely and efficiently in an interactive environment is challenging. A large amount of research has been devoted to improve the intelligence of a UAV while performing a mission, where finding an optimal maneuver decision-making policy of the UAV has become one of the key issues when we attempt to enable the UAV autonomy. In this paper, we propose a maneuver decision-making algorithm based on deep reinforcement learning, which generates efficient maneuvers for a UAV agent to execute the airdrop mission autonomously in an interactive environment. Particularly, the training set of the learning algorithm by the Prioritized Experience Replay is constructed, that can accelerate the convergence speed of decision network training in the algorithm. It is shown that a desirable and effective maneuver decision-making policy can be found by extensive experimental results.

## 1. Introduction

With the development of the control and electronic techniques in recent years, the performance of unmanned aerial vehicle (UAV) has been improved rapidly in all aspects. The UAV has been applied to assisting, replacing people to complete difficult missions due to high mobility, great flying height, and low cost [1]. Thus, it is necessary to improve the autonomy of UAV while performing some special airdrop tasks without risking human lives, such as delivery of relief supplies [2], extinguishing by UAV, and so on. Consequently, how to improve the autonomous flight capability of UAV becomes the research focus of researchers in various countries [3].

At present, the airdrop tasks are typically implemented incorporating with path panning [4], which is a method for searching an optimal path from start point to end point while avoiding obstacles in the environment. Conventional path planning algorithms for UAVs include Visibility Graph [5,6]; randomly sampling search algorithms, like Rapidly-exploring Random Tree [7], Probabilistic Roadmap [8]; heuristic algorithms, such as A-Star [9], Sparse A-Star (SAS) [10], and D* [11]; and genetic algorithms [12]. Then, a UAV can fly to target point by following the planned route, where various trajectory tracking algorithms are proposed [13,14]. However, this kind of schemes have some disadvantages. For example, an optimal route relies on a priori knowledge about the environment, in which the data of terrain and obstacles is usually difficult to obtain, limiting our capability of environment modelling. Moreover, when the environment becomes dynamic, involving moving obstacles, these schemes are not flexible enough to alter their control strategies immediately. A replan of paths have to be scheduled to adapt to the changes in the environment. Therefore, it is desired to design an end-to-end algorithm that can manipulate a UAV to flight autonomously in a dynamic environment without path planning and trajectory tracking.

A promising direction is inspired by the AlphaGo developed by Google based on deep reinforcement learning, which can play Atari games using a kind of end-to-end decision-making algorithm, called Deep Q Network (DQN) [15]. The performance of this algorithm reached human level after an extensive training, which shows the potential of the reinforcement learning-based methods combined with deep learning in solving practical problems. Meanwhile, in order to solve the dimension explosion caused by the continuity of action space, the Deep Deterministic Policy Gradient (DDPG) was proposed in Reference [16], and the experience replay is used in these Deep Reinforcement Learning (DRL)-based algorithms and allow agents to remember and learn from historical data. The DDPG overcomes the dimension explosion issue caused by continuous action space and state space. However, it forms training set by taking samples from data memory with the Uniform Experience Replay (UER), which does not fully exploit the diversity of historical data. Moreover, the UER usually has a low convergence rate of neural network and even divergence in training neural networks. Therefore, the Prioritized Experience Replay (PER) was proposed to improve the efficiency of learning from experiences [17]. In this paper, we construct a new priority of each experience based on Double Q-Learning [18], which could overcome convergence fluctuation caused by over estimate compared with Reference [19].

In the present work, we aim to tackle the challenges mentioned above and focus on the UAV maneuver decision-making for airdrop task. The main works presented in this paper are summarized as follows:

- The UAV Maneuver Decision-Making Model for airdrop tasks is built based on Markov Decision Processes (MDPs). Particularly, we design the flight state space, the flight action space, and the reward functions. Among the components of model, we devote our air-to-ground drop theory to designing and constructing the UAV maneuver decision-making model for airdrop tasks.
- We propose the Maneuver Decision-Making Algorithm for autonomous airdrop based on DDPG with PER sampling method (PER-DDPG) to train a UAV for generating efficient maneuver under target point constraint refined from the model we designed in an interactive environment. Specially, we design the decision-making function by deep neural network and construct the training set sampling method based on PER.
- Simulation results show that the algorithm we proposed could improve the autonomy of a UAV during the airdrop task and the PER is able to accelerate the efficiency of learning from experiences. Moreover, we find that the winning rate of the PER-based algorithm exceeds the UER-based algorithm 2–4%.

This paper is organized as follows: Section 2 describes the background knowledge of all the methods used to design UAV maneuver decision-making model and algorithm. Section 3 presents the details of experimentations we designed, comparison of learning rate and winning rate under UER and PER sampling methods separately. Section 4 shows the conclusion of our work and looks forward to the future of our research.

## 2. Methodology

The UAV has been used to help people finish some dangerous and repetitive missions, such as crop protection, wildlife surveillance, traffic monitoring, electric power inspection, search, and rescue operations. A need for more advanced and simple UAV autonomous flight solution has emerged. As mentioned above, traditional solution of real time obstacle avoidance for manipulators and UAVs is that algorithm plans an optimal path and then UAV follows path by trajectory tracking method. In this paper, we redefined the process of UAV autonomous flight and constructed the UAV Maneuver Decision-Making Model for Airdrop Task based on MDPs [20]. On the other hand, we proposed a novel UAV Maneuver Decision-Making algorithm based on Deep Reinforcement Learning [21].

**Figure 1:**
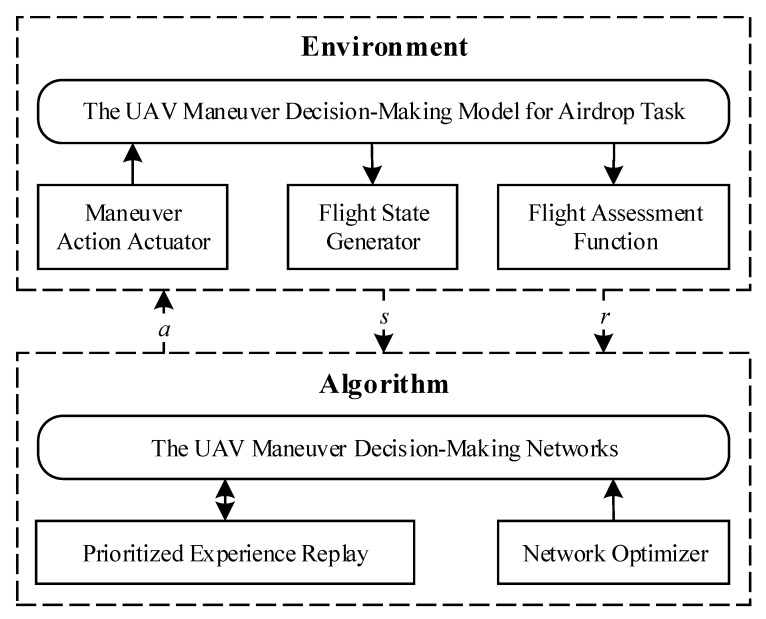
The structure of unmanned aerial vehicle (UAV) Maneuver Decision-Making Algorithm.

As shown in Figure 1, we construct the UAV Maneuver Decision-Making Model for Airdrop Task Based on MDPs firstly. Among this model, we design the flight action space, the flight state space and flight assessment function that are used to demonstrate the characteristics of UAV autonomous flight during airdrop. Moreover, we design and realize the UAV Maneuver Decision-Making Algorithm, including the UAV maneuver decision-making network, prioritized experience replay used to sample training data from historical experiences, and network optimizer applied to train networks.

### 2.1. The UAV Maneuver Decision-Making Model for Airdrop Task Based on Mdps

#### 2.1.1. Markov Decision Processes

During the process of performing airdrop task, UAV maneuver decision-making could be regarded as a sequential decision process. Moreover, while selecting optimal action for UAV, the controller usually considers current information from environment. Thus, we can think that this decision process is Markovian and could use MDPs to model the UAV maneuver decision-making model for airdrop task.

MDPs can be described by a quintuple

(1) {T,S,A(s),P(·|s,a),R(s,a)},

where *T* represents the decision time, *S* represents the system state space, A(s) represents the system action space, and the transition probability P(·|s,a) represents the probability distribution of the system at the next moment when the system used the action a∈A(s) in the state s∈S. The reward function R(s,a) represents the benefit that the decision-maker gets when the action a∈A(s) is taken in the state s∈S. Based on the MDPs, we can define a complete mathematical description of the UAV Airdrop Task.

**Figure 2:**
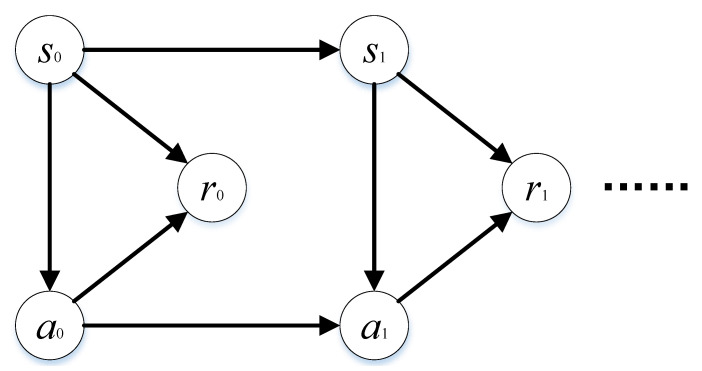
The structure of finite Markov Decision Processes (MDPs).

As shown in Figure 2, MDPs can be summarized as follows: the initial state $s_{0}$ of the system is that the decision-maker chooses action $a_{0}$ and executes it, system moves to next state $s_{1}$ according to a certain transition probability $P(\cdot |s_{0},a_{0})$, and so on. In this process, the decision-maker earned rewards sequence $\left(r_{0},r_{1},\cdots \right)$. Among this process, the decision-maker is stimulated by external rewards, and rewards are maximized by constantly updating policy. The action adopted by decision-maker is a=μ(s), where μ(s) is effective policy, and the utility function (at state s∈S, the expected reward obtained by adopting the policy μ) is v(s,μ). When current policy is the optimal policy, Equation (2) should be satisfied.

(2) $$v\left(s\right)=\underset{\mu }{\sup}v\left(s,\mu \right),s\in S.$$

Based on the characteristics of UAV maneuver decision-making for airdrop, we use infinite stage discount model as utility function, as shown in Equation (3).

(3) $$v\left(s,\pi \right)=\sum_{t=0}^{\infty }\gamma^{t}E_{\mu }^{s}\left[R\left(s_{t},a_{t}\right)\right],s\in S.$$

In the equation above, γ∈[0,1] is the future reward discount factor. $E\left[\cdot \right]$ represents mathematical expectation; thus, Equation (3) indicates that the expected objective of discount model is the sum of expectation of reward multiplied by a discount factor at every decision moment *t*. Thereby, the optimal policy under the discount model can be obtained by lots of iterations.

In the following, we will demonstrate the problems definition among Airdrop Task firstly. Then, state space *S*, action space A(s), transition probability model P(·|s,a), and reward function R(s,a) will be designed.

#### 2.1.2. Problems Definition among Airdrop Task

**Figure 3:**
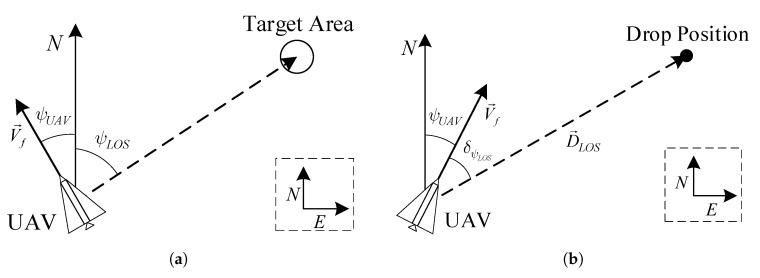
Problems Description among UAV Airdrop Task: (**a**) the UAV should turn its nose towards target area firstly; (**b**) after adjustment of azimuth, the UAV could follow the guidance of UAV maneuver decision-making policy until reaching target position.

Before we start running a Reinforcement Learning (RL)-based algorithm, we should construct a simulation model of problems to be solved. Thus, we define two problems that usually occur during airdrop task. Generally, when the UAV prepares to perform airdrop task, it should turn nose towards target area firstly, and then fly to target position by following the UAV maneuver decision-making policy, as shown in Figure 3. In Figure 3a, *N* and *E* represent North and East directions, and ${\vec{V}}_{f}$ and $\psi_{UAV}$ are the velocity and azimuth of UAV separately. Moreover, the dashed line between UAV and Target Area indicates the expected azimuth of UAV, that is expressed by $\psi_{LOS}$. Figure 3b, the drop position of UAV is a solid point, and ${\vec{D}}_{LOS}$ and $\delta_{\psi_{LOS}}$ represents Line of Sight (LOS) and azimuth of LOS between UAV and Target Position. Therefore, two problems involved in the airdrop task:

- Turn round problem: UAV flies from a random starting point and turns to the direction of target area demanded. During this process, pilots usually manipulate UAVs and controls the azimuth of UAVs towards target direction.
- Guidance problem: UAV starts from a random position and flies to a drop position given by commander. If the pilot want to manually finish this work, it will take lots of energy because the pilot should plan an effective path and manipulate the UAV following it.

If we want to simulate this process described above, a dynamical model of UAV should be constructed. We adopted a dynamical model describing airdrop task based on 3-DoF kinematic model of UAV [22]. When the position and attitude of UAV are confirmed at t, we can obtain the state of UAV at t+1 by solving the model we designed. Therefore, we think that the transition probability of UAV maneuver decision-making model is $P\left(\cdot |s,a\right)=1$, which belongs to deterministic model.

Based on the 3-DoF kinematic model of UAV, the flight state is defined as $\left(x,z,v,\psi_{c}\right)$, where $\left(x,z\right)$ is the horizontal coordinates of the UAV in the geographical coordinate system, and $\psi_{c}$ is the flight path azimuth Angle of the UAV. On the other hand, the steering overload of UAV is defined as $N_{s}\in \left[-N_{y}^{max},N_{y}^{max}\right]$, where $N_{y}^{max}$ represents the max normal acceleration of UAV in the body coordinate system. During the simulation process, algorithm outputs the current optimal maneuver control $N_{s}$ and the next state ${\left(x,z,v,\psi_{c}\right)}^{\prime }$ of UAV is calculated combined with the current state $\left(x,z,v,\psi_{c}\right)$ according to the flight simulation model of UAV.

#### 2.1.3. State Representation and Action Design

Based on the problems’ definition among airdrop task, we can design the state space of two problems mentioned above separately. Specifically, action space is the same for both problems because the core kinematic model is all realized based on the 3-DoF kinematic model of UAV.

##### (1) State Space of Turn Round Problem

Considering that turn round problem is related to the azimuth of UAV and the relative direction of relative orientation between UAV and target area, we designed the state space of turn round problem, that is defined as

(4) $$S=\left\{\delta_{\psi_{LOS}},N_{s}\right\},$$

where $\delta_{\psi_{LOS}}$ is the relative azimuth between LOS and nose direction of UAV, and $N_{s}$ represents the steering overload of UAV. $\delta_{\psi_{LOS}}$ could be calculated by

(5) $$\delta_{\psi_{LOS}}=\psi_{LOS}-\psi_{UAV},$$

where $\psi_{LOS}$ is the azimuth of LOS relative to North, and $\psi_{UAV}$ is the azimuth of UAV relative to North. Two symbols are all in $\left[0,2\pi \right]$ and satisfy the right-hand rule.

##### (2) State Space of Guidance Problem

For the guidance problem of airdrop task, we can define its state space as

(6) $$S=\left\{d_{LOS},\delta_{\psi_{LOS}},N_{s}\right\},$$

where $d_{LOS}$ indicates the distance between UAV and drop position. Moreover, if we define ${\vec{X}}_{UAV}$ as the position of UAV and ${\vec{X}}_{TGT}$ as the drop position, we will calculate the symbols by

(7) $$\left\{\begin{array}{c} d_{LOS}={∥{\vec{D}}_{LOS}∥}_{2}={∥{\vec{X}}_{TGT}-{\vec{X}}_{UAV}∥}_{2}\\ \delta_{\psi_{LOS}}=\arccos\left(\frac{{\vec{V}}_{f}\cdot {\vec{D}}_{LOS}}{\left|{\vec{V}}_{f}\right|\cdot \left|{\vec{D}}_{LOS}\right|}\right) \end{array},\right.$$

where ${∥\cdot ∥}_{2}$ represents the 2-norm of vector.

##### (3) Action Space of Both Problems

Based on the flight simulation model of UAV we constructed, we can establish the action space as below.

(8) $$A\left(s\right)=\left\{N_{s}\right\}.$$

#### 2.1.4. Reward Function Based on Potential-Based Reward Shaping

In MDPs, the reward function determines the direction of policy iteration and directly reflects the agent’s intention. The termination condition of turn round problem can be defined as follows:

(9) $$\left|\delta_{\psi_{LOS}}^{t}\right|\leq \delta_{\psi }^{\min},$$

where $\delta_{\psi_{LOS}}^{t}$ is $\delta_{\psi_{LOS}}$ at decision moment *t*, and $\delta_{\psi }^{min}$ is the minimum error of $\delta_{\psi_{LOS}}$. This termination condition means that, when the UAV heads to the target area with an error under certain tolerance, we could think that the turn round problem has been solved. Analogously, the termination condition of guidance problem could be also obtained as

(10) $${∥{\vec{D}}_{LOS}^{t}∥}_{2}\leq d_{LOS}^{\min},$$

where ${\vec{D}}_{LOS}^{t}$ is ${\vec{D}}_{LOS}$ at decision moment *t*, and $d_{LOS}^{min}$ is the minimum error of the distance between the UAV and the required drop position.

Therefore, the reward function R(s,a) of the problems could be defined as

(11) $$R\left(s,a\right)=\left\{\begin{array}{cc} 1.0, & \mathrm{Satisfy}\;\mathrm{Termination}\;\mathrm{Condition}\\ 0.0, & \mathrm{Not}\;\mathrm{Satisfy}\;\mathrm{Termination}\;\mathrm{Condition} \end{array}.\right.$$

Equation (11) indicates that if UAV’s state satisfies termination condition of problems, R(s,a) will return 1.0, otherwise 0.0.

The algorithm we proposed could search the optimal policy according to this kind of episodic reward, but there is a serious fault that is possible to influence the efficiency of policy convergence because the rewards environment returned are too sparse to learn useful experience, such as those samples whose reward is not zero. Thus, some researchers proposed the potential-based reward shaping [23,24] (PBRS) method to solve the problem brought by “sparse” reward. PBRS provides a guidance signal for improving the speed of policy convergence by adding a new reward shaping function $F(s,a,s^{\prime })$ to original reward function. Generally, $F(s,a,s^{\prime })$ should satisfy

(12) $$F\left(s,a,s^{\prime }\right)=\gamma \Phi \left(s^{\prime }\right)-\Phi \left(s\right),$$

where $\gamma \in \left[0,1\right]$ is a discount factor, and s∈S, a∈A(s), and $s^{\prime }$ indicate the current state, the current action, and the next state, respectively. Moreover, Φ(s) is a kind of potential energy function. If agent’s action help it approach termination condition, it will receive a positive reward, otherwise negation. In summary, traditional MDPs can be rewritten as

(13) $$\left\{T,S,A\left(s\right),P\left(\cdot |s,a\right),R\left(s,a,s^{\prime }\right)\right\},$$

and new reward function is defined as

(14) $$R\left(s,a,s^{\prime }\right)=R\left(s,a\right)+F\left(s,a,s^{\prime }\right).$$

In the following, we present the shaping function for each individual problem.

##### (1) The Shaping Function for Turn Round Problem

Based on the definition of turn round problem, we can obtain the shaping function for turn round problem as shown in Equation (15).

(15) Fs,a,s′=ωδψLOS·π−δψLOSπ+ωNs·1−Nymax−NsNymax+1Nymax−NsNymax+12/2−2π−δψTGT+π2π.

In Equation (15), $\omega_{\delta_{\psi_{LOS}}}\in \left[0,1\right]$ is the coefficient of azimuth error factor, and $\omega_{N_{s}}\in \left[0,1\right]$ is the coefficient of maneuver factor.

##### (2) The Shaping Function for Guidance Problem

As shown in Equation (16), the definition of the shaping function of guidance problem is given.

(16) Fs,a,s′=ωδdT·δdLOSvmax·T+1δdLOSvmax·T+12/2+ωNs·1−Nymax−NsNymax+1Nymax−NsNymax+12/2−2π−δψLOS+π2π.

In the equation, $\delta_{d_{LOS}}$ is distance that UAV approaches drop position after one simulation step, $\omega_{\delta_{d_{LOS}}}\in \left[0,1\right]$ is the coefficient of distance factor. Moreover, *T* is simulation step, $v_{max}$ is the maximum speed of UAV. The symbol $\delta_{d_{LOS}}$ could be calculated by

(17) $$\delta_{d_{LOS}}=d_{LOS}^{t}-d_{LOS}^{t+1},$$

where $d_{LOS}^{t}$ is the distance between UAV and drop position at *t*-th decision step.

### 2.2. The UAV Maneuver Decision-Making Algorithm for Autonomous Airdrop Based on Per-DDPG

#### 2.2.1. The Framework of Per-DDPG

The Deep Deterministic Policy Gradient (DDPG) is a model-free, off-policy, and DRL-based algorithm based on Actor-Critic architecture. It can effectively solve problems belonging to MDPs with continuous state space and action space.

Although DDPG can avoid the dimensional explosion problem brought by continuous state space and action space, it does not consider the diversity of data and does not utilize historical experience fully. This results in the low convergence speed of DDPG’s policy and poor stability of the convergence result. Meanwhile, because the episode of UAV maneuver decision-making is short, while the task process lasts a long time, the changing of reward is not obvious. Thus, the value density of historical experience is low. That is the reason why we use PER to generate training data [17], which can improve the utilization of the potential value of historical experiences, thereby increasing convergence speed and enhancing the stability of training results.

**Figure 4:**
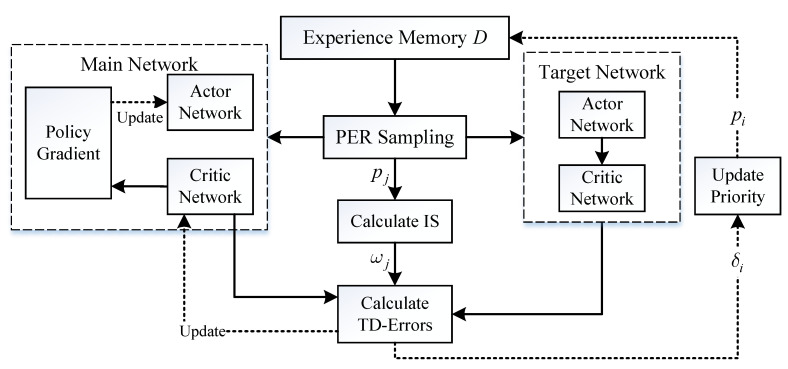
The diagram of Prioritized Experience Replay-Deep Deterministic Policy Gradient (PER-DDPG)’s framework.

Figure 4 shows the block diagram of the PER-DDPG’s structure. At each decision-making step, the actor network outputs action with noise for exploring according to state, and the current state, action, reward, and next state are packaged and stored in experience memory *D*. During the process of storing experience, samples bind with probability used for PER sampling. And then, the training data is sampled from *D* by PER, and every sampled data’s TD-error [25] between current Q(s,a) and target value is calculated for updating the priority of data and being cumulated for updating network’s parameters with importance sampling (IS) weights. Finally, the parameters of main networks $Q(s,a;\theta^{Q})$ and $\mu (s;\theta^{\mu })$ are updated, and the parameters of target networks $Q(s,a;\theta^{Q^{\prime }})$ and $\mu (s;\theta^{\mu^{\prime }})$ are also updated smoothly because of stability of network training.

At each moment, the algorithm gives action by

(18) $$a_{t}=\mu \left(s_{t}\right),$$

where $s_{t}\in S$ is the current state, and $a_{t}$ is the resulting output by the actor function μ(s). During the training process, the critic function Q(s,a) evaluates current action given by actor function, and the evaluation is used for the basis of updating μ(s).

#### 2.2.2. The UAV Maneuver Decision-Making Network

As mentioned above, DDPG is a kind of deep reinforcement learning algorithm based on the Actor-Critic framework. During the training process, the actor network outputs action a∈A(s) according to state s∈S generated by environment. Meanwhile, TD-error is used to optimize the critic network and update its parameters. Similarly, the actor network’s parameters are optimized according to maxQ(s,a). Therefore, we must design the structure of actor and critic networks, respectively, on the basis of DL.

##### (1) Actor Network

**Figure 5:**
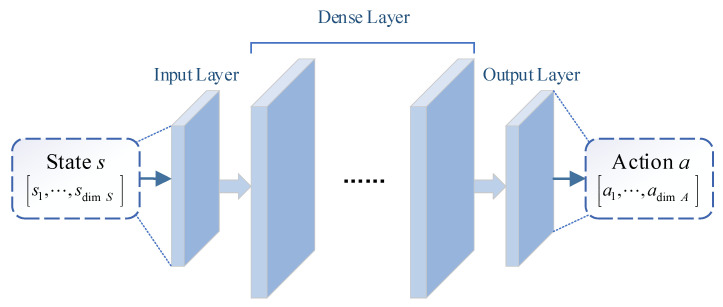
The structure of actor network.

The actor network $\mu (s;\theta^{\mu })$ is mainly used to output action in real-time decision according to state. The input vector of the network is the current state s∈S, and the output vector of the network is the current action a∈A(s) calculated by $\mu (s;\theta^{\mu })$. Considering the definition of state space, the dimension of network input is dim(S), and the dimension of output is dim(A). As shown in Figure 5, it is the normal structure of actor network $\mu (s;\theta^{\mu })$.

##### (2) Critic Network

**Figure 6:**
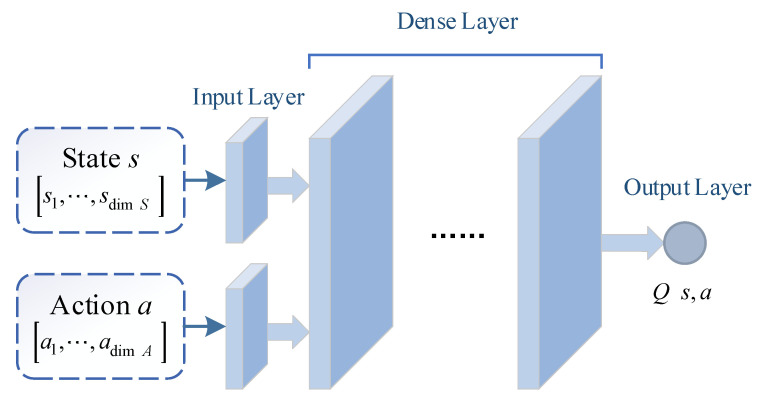
The structure of critic network.

The critic network $Q(s,a;\theta^{Q})$ is used to evaluate the advantage of current action a∈A(s) output by $\mu (s;\theta^{\mu })$. The network input is [s,a], and the network output is Q(s,a). According to state space and action space defined above, the dimension of network input is dim(S)+dim(A), and the dimension of network output is 1. As shown in Figure 6, it is the normal structure of critic network $Q(s,a;\theta^{Q})$.

In addition, before state and action are entered into network, the value of input vector should be normalized for eliminating the influence of data’s physical meaning. Moreover, the structure of target networks $\mu^{\prime }\left(s;\theta^{\mu^{\prime }}\right)$ and $Q^{\prime }\left(s,a;\theta^{Q^{\prime }}\right)$ is similar to $\mu (s;\theta^{\mu })$ and $Q(s,a;\theta^{Q})$, and only the method of parameters updating is distinguished.

#### 2.2.3. The Training Procedure of UAV Maneuver Decision-Making Algorithm

Based on MDPs, the key issue of searching optimal UAV maneuver decision-making policy is to solve an optimization problem defined as

(19) $$\max_{\pi }Q\left(s,a\right)=E_{\mu }\left[v\left(s,\pi \right)\right],$$

where v(s,μ) is defined in Equation (3). In this paper, we use Double Q-Learning [18] to update the Q(s,a), as defined in Equation (20).

(20) $$Q\left(s,a\right)=Q\left(s,a\right)+\sigma \left[r+\gamma Q^{\prime }\left(s^{\prime },\underset{a}{\arg\max}Q\left(s^{\prime },a\right)\right)-Q\left(s,a\right)\right].$$

In the equation above, s∈S is current state, a∈A(s) is current action, $r=R(s,a,s^{\prime })$ is current reward, $s^{\prime }\in S$ is next state, and σ∈[0,1] is the learning rate of the algorithm. As shown in Equation (21), it is loss function $L(\theta^{Q})$ of critic network $Q(s,a;\theta^{Q})$.

(21) $$L\left(\theta^{Q}\right)=E_{{\left(s,a,r,s^{\prime }\right)}_{j}}\left[{\delta_{j}}^{2}\right].$$

The symbol $\delta_{j}$ is TD-error based on Double Q-Learning of *j*-th data sampling from memory *D*. TD-error describes the difference between $Q(s,a;\theta^{Q})$ and optimal goal, and it is defined as

(22) $$\delta_{j}=y_{j}-Q\left(s_{j},a_{j};\theta^{Q}\right),$$

where $y_{j}$ is the optimal goal of $Q(s_{j},a_{j};\theta^{Q})$, ${\left(s,a,r,s^{\prime }\right)}_{j}$ is *j*-th training data, and $s_{j}$ and $a_{j}$ are current state and action in ${\left(s,a,r,s^{\prime }\right)}_{j}$, respectively. The symbol $y_{j}$ could be calculated by

(23) $$y_{j}=\left\{\begin{array}{cc} r_{j}, & \mathrm{if}s_{j}\;\mathrm{is}\;\mathrm{Termination}\;\mathrm{State}\\ r_{j}+\gamma Q^{\prime }\left(s_{j+1},\mu^{\prime }\left(s_{j+1};\theta^{\mu^{\prime }}\right);\theta^{Q^{\prime }}\right), & \mathrm{Otherwise} \end{array},\right.$$

where $r_{j}$ and $s_{j+1}$ are current reward and next state in ${\left(s,a,r,s^{\prime }\right)}_{j}$. Thus, we can obtain the gradient of loss function $L(\theta^{Q})$ as shown below considering Equations (21) and (22).

(24) $$\nabla_{\theta^{Q}}L\left(\theta^{Q}\right)=E_{{\left(s,a,r,s^{\prime }\right)}_{j}}\left[\delta_{j}\cdot \nabla_{\theta^{Q}}Q\left(s_{j},a_{j};\theta^{Q}\right)\right].$$

At the same time, we define the loss function $L(\theta^{\mu })$ of actor network $\mu (s;\theta^{\mu })$ in order to update the parameters of $\mu (s;\theta^{\mu })$.

(25) $$L\left(\theta^{\mu }\right)=E_{s}\left[Q\left(s,\mu \left(s;\theta^{\mu }\right);\theta^{Q}\right)\right].$$

Thereby, we can obtain the gradient of $L(\theta^{\mu })$ according to deterministic policy gradient theorem [26], as shown in Equation (26).

(26) $$\nabla_{\theta^{\mu }}L\left(\theta^{\mu }\right)=E_{s}\left[\nabla_{\theta^{\mu }}\mu \left(s;\theta^{\mu }\right)\nabla_{a}{\left.Q\left(s,a;\theta^{Q}\right)\right|}_{a=\mu \left(s;\theta^{\mu }\right)}\right].$$

During the process of training networks, we use the PER method to sample training data from *D* in order to utilize the diversity of experiences fully. Usually, the training data is sampled by selecting a batch of data from *D* uniformly, which means the probability P(i) of each sample selected in *D* is equal. On the contrary, P(i) of PER is not same, as defined as Equation (27).

(27) $$P\left(i\right)=\frac{p_{i}^{\alpha }}{\sum_{k}p_{k}^{\alpha }.}$$

In the equation above, $p_{i}$ is the priority of *i*-th sample in *D*, and α is a hyperparameter. When α=0, it is pure UER. $p_{i}$ is defined based on TD-error, as shown in Equation (28).

(28) $$p_{i}=\left|\delta_{i}\right|+\epsilon .$$

Among the equation above, $\delta_{i}$ is TD-error of *i*-th sample in *D*. Moreover, a minimum ϵ≤0.0001 is introduced to prevent $p_{i}$ from being 0.

Although PER improves the availability of experiences, the distribution error of training data sampled by PER occurs compared with UER’s, and this problem also reduces the diversity of training samples. Therefore, importance sampling (IS) weights are introduced to correct the distribution error of training data caused by PER. The IS weight $\omega_{j}$ is defined as Equation (29).

(29) $$\omega_{j}={\left(\frac{1}{N}\cdot \frac{1}{P\left(j\right)}\right)}^{\beta }.$$

In the equation above, *N* is the size of *D*. When β=1, the distribution error of training set is fully compensated. When $\delta_{j}$ is calculated, the actual updating target is $\omega_{j}\cdot \delta_{j}$ and it’s used to replace $\delta_{j}$ in Equation (24). Therefore, the final gradient Δ of $Q(s_{j},a_{j};\theta^{Q})$ is calculated by

(30) $$\Delta =\sum_{j}\omega_{j}\cdot \delta_{j}\cdot \nabla_{\theta^{Q}}Q\left(s_{j},a_{j};\theta^{Q}\right).$$

In order to ensure the stable convergence of the network, $\omega_{j}$ is normalized by $\frac{\omega_{i}}{\max_{j}\omega_{j}}$. Thereby, the actual IS weight $\omega_{j}$ could be defined as

(31) $$\omega_{j}={\left(\frac{\min_{i}P\left(i\right)}{P\left(j\right)}\right)}^{\beta }.$$

At the same time, in the early stage of training, the distribution error caused by PER is not big. Thus, we define an initial $\beta_{0}\in \left(0,1\right)$, and it gradually increases to 1 with training going on.

In addition, because of stability of target networks’ training, the parameters of $\mu^{\prime }\left(s;\theta^{\mu^{\prime }}\right)$ and $Q^{\prime }\left(s,a;\theta^{Q^{\prime }}\right)$ are updated by “Soft” updating similar to smooth updating, as shown in Equation (32).

(32) $$\left\{\begin{array}{c} \theta^{Q^{\prime }}=\tau \theta^{Q}+\left(1-\tau \right)\theta^{Q^{\prime }}\\ \theta^{\mu^{\prime }}=\tau \theta^{\mu }+\left(1-\tau \right)\theta^{\mu^{\prime }} \end{array}.\right.$$

In the equation above, the symbol τ∈(0,1) is a hyperparameter involved in the “Soft” updating. Moreover, a kind of random noise is used to improve the exploration ability of deterministic policy involved in algorithm, as shown in Equation (33).

(33) $$a_{t}=\mu \left(s_{t};\theta^{\mu }\right)+N\left(t\right).$$

Among the equation, N(t) is a kind of time-variant noise. Because the UAV maneuver decision-making satisfies the Markovian condition and the changing of state is inertial process, an autocorrelation noise model called Ornstein Uhlenbeck (OU) process [27] is used for action exploration. The iterative formula of N(t) is shown in Equation (34).

(34) $$x_{t+\Delta t}=x_{t}+\kappa \cdot \left(\mu -x_{t}\right)\cdot \Delta t+dW_{t},dW_{t}∼N\left(0,\sigma^{2}\Delta t\right).$$

In the equation above, $x_{t}$ and $x_{t+\Delta t}$ are current and next value of noise separately. μ and κ indicate the mean value and regression rate of noise, respectively. Moreover, Δt is the step of noise, and $dW_{t}$ represents the Wiener process.

Finally, the training procedure of UAV maneuver decision-making algorithm is given in Algorithm 1.
**Algorithm 1** The UAV Maneuver Decision-Making Algorithm for Airdrop Task.**Input:****The hyperparameters of training networks:** the size of minibatch *k*, networks’ learning rate η;**The hyperparameters of updating policy:** policy’s learning rate σ, learning period *K*, memory capacity *N*, “Soft” updating τ;**The hyperparameters of sampling:** the availability exponent of PER α, IS exponent β;**The control parameters of simulation:** maximum period *M*, maximum step per period *T*.**Output:**Actor network $Q(s_{j},a_{j};\theta^{Q})$ and its target network $Q^{\prime }\left(s,a;\theta^{Q^{\prime }}\right)$;Critic network $\mu \left(s;\theta^{\mu }\right)$ and its target network $\mu^{\prime }\left(s;\theta^{\mu^{\prime }}\right)$.1:Initialize $Q(s_{j},a_{j};\theta^{Q})$, $\mu \left(s;\theta^{\mu }\right)$ and their target networks $Q^{\prime }\left(s,a;\theta^{Q^{\prime }}\right)$, $\mu^{\prime }\left(s;\theta^{\mu^{\prime }}\right)$.2:**for**m=1 to *M* **do**3: Reset environment and read the initial state $s_{0}$.4: Output $a_{0}$ according to Equation (18).5: **for** t=1 to *T* **do**6:  Observe current state $s_{t}$ and reward $r_{t}$ of environment and calculate current action $a_{t}$ according to Equation (18).7:  Save current transition $\left(s_{t},a_{t},r_{t},s_{t+1}\right)$ into experiences memory *D*.8:  **if** tmodK≡0 **then**
9:   Reset the gradient Δ=0 of $Q(s_{j},a_{j};\theta^{Q})$ with IS.10:   **for** j=0 to *k* **do**11:    Sample traing data $j∼P\left(j\right)$ according to Equation (27)12:    Calculate IS weight $\omega_{j}$ according to Equation (31)13:    Calculate TD-error $\delta_{j}$ of training data according to Equation (22) and update its priority according to Equation (28)14:    Accumulate Δ according to Equation (30).15:   **end for**16:   Update the parameters of $Q(s_{j},a_{j};\theta^{Q})$ according to Δ with learning rate η.17:   Update the parameters of $\mu \left(s;\theta^{\mu }\right)$ according to Equation (26).18:   Update the parameters of target networks $Q^{\prime }\left(s,a;\theta^{Q^{\prime }}\right)$ and $\mu^{\prime }\left(s;\theta^{\mu^{\prime }}\right)$ according to Equation (32)19:  **end if**20: **end for**21:**end for**

## 3. Results and Analysis

According to content aforementioned, we design some experiments to verify the availability of the algorithm we proposed and compare PER with UER in terms of the efficiency of policy optimization. In the following, we will explain the setting of simulation environment, training results, and results of Monte-Carlo (MC) test experiments, as well as their analysis.

### 3.1. The Settings of Simulation Environment

In the experiments we designed, the drop area and UAV are restricted to 100 km × 100 km airspace at the height of 5000 m. For each simulation experiment, the UAV’s initial state is randomly generated, and the UAV might start from arbitrary position in flight airspace. In order to make simulation closer to real environment, we decide to make T=0.5 s because UAV’s control input is usually updated by human pilot every 0.5 s ∼ 1 s.

Moreover, because each dimension of state space has different physical units, the state and action should be normalized before it’s input into $Q(s,a;\theta^{Q})$ and $\mu (s;\theta^{\mu })$. As shown in Table 1, the details of data are explained. Thereby, we can normalize parameters according to their physical meanings.

### 3.2. The Simulation Results and Analysis of Turn Round Problem

#### 3.2.1. The Parameters Setting of Algorithm

According to the training procedure of algorithm, before we start training, some parameters should be assigned. As shown in Table 2, there are some parameters assignments of algorithm. Moreover, we design the structure of networks $\mu (s;\theta^{\mu })$ and $Q(s,a;\theta^{Q})$ shown in Table 3 and Table 4, respectively, according to the state space and action space of turn round problem. In this paper, the networks are all designed by fully-connected neural network, which means the layers are dense layers.

#### 3.2.2. The Analysis of Simulation Results

**Figure 7:**
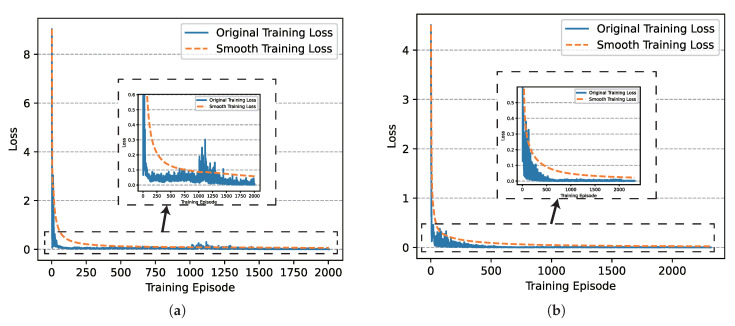
The comparison of critic networks’ training loss involved in Uniform Experience Replay (UER)-DDPG and PER-DDPG over training episode. (**a**) The training loss of critic network of UER-DDPG. (**b**) The training loss of critic network of PER-DDPG.

Based on the setting above, we finished the training of networks successfully and the loss diagrams of critic networks involved in UER-DDPG and PER-DDPG over time are shown in Figure 7, respectively. We could find that the loss of PER-DDPG converges faster than UER-DDPG. Moreover, the loss of PER-DDPG becomes stable after converging to minimum. On the contrary, when UER-DDPG converges, the loss fluctuated greatly at 1000th episode, and its convergence costs more time.

**Figure 8:**
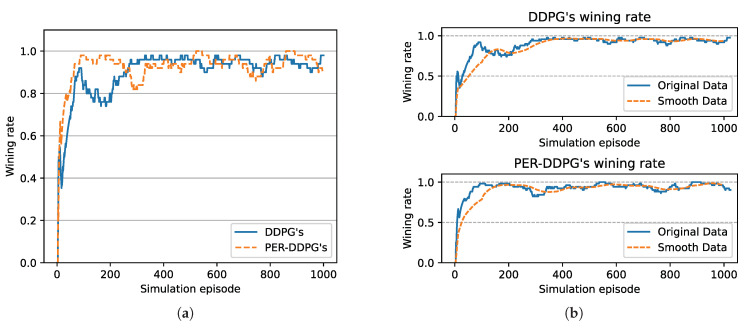
The winning rate of algorithms based on UER-DDPG and PER-DDPG over simulation episode. Winning rate means a rate of finishing mission successfully. (**a**) The comparison of winning rate based on UER-DDPG and PER-DDPG. (**b**) The top figure is the winning rate of algorithm based on UER-DDPG, and the figure at the bottom is the winning rate of algorithm based on PER-DDPG.

**Figure 9:**
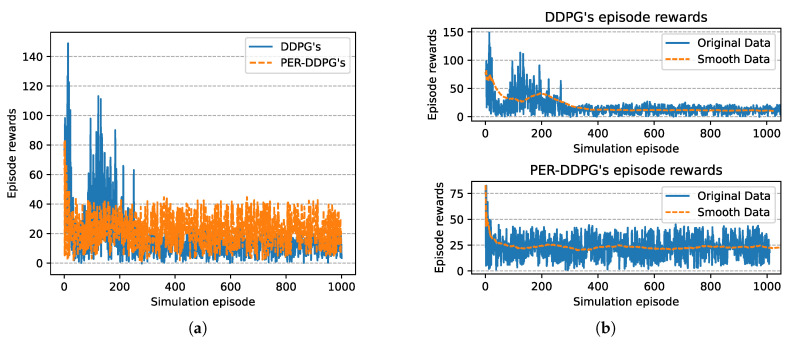
The episode rewards of algorithms based on UER-DDPG and PER-DDPG over simulation episode. (**a**) The comparison of episode rewards based on UER-DDPG and PER-DDPG. (**b**) The top figure is the episode rewards of algorithm based on UER-DDPG, and the bottom figure is the episode rewards of algorithm based on PER-DDPG.

Figure 8 is the winning rate of algorithms based on different experience replay methods over simulation episode during the training process. We can find that all the winning rates are more than 80% and maintain stably. It is shown that DDPG with PER method could achieve the same result compared with UER-DDPG. But the training process of PER-DDPG is much stabler than UER-DDPG because the winning rate of UER-DDPG fluctuates violently at the beginning of training. Meanwhile, the curve of episode rewards further demonstrates that PER-DDPG is much steadier than UER-DDPG from Figure 9.

**Figure 10:**
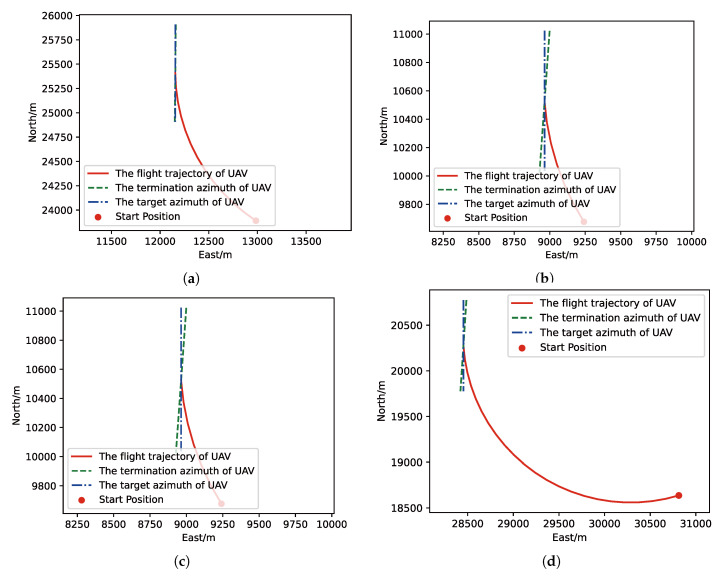
The flight trajectory from Monte-Carlo (MC) experiments for the trained result of UER-DDPG: (**a**) the flight trajectory of 1st experiment; (**b**) the flight trajectory of 2nd experiment; (**c**) the flight trajectory of 3rd experiment; (**d**) the flight trajectory of 4th experiment.

**Figure 11:**
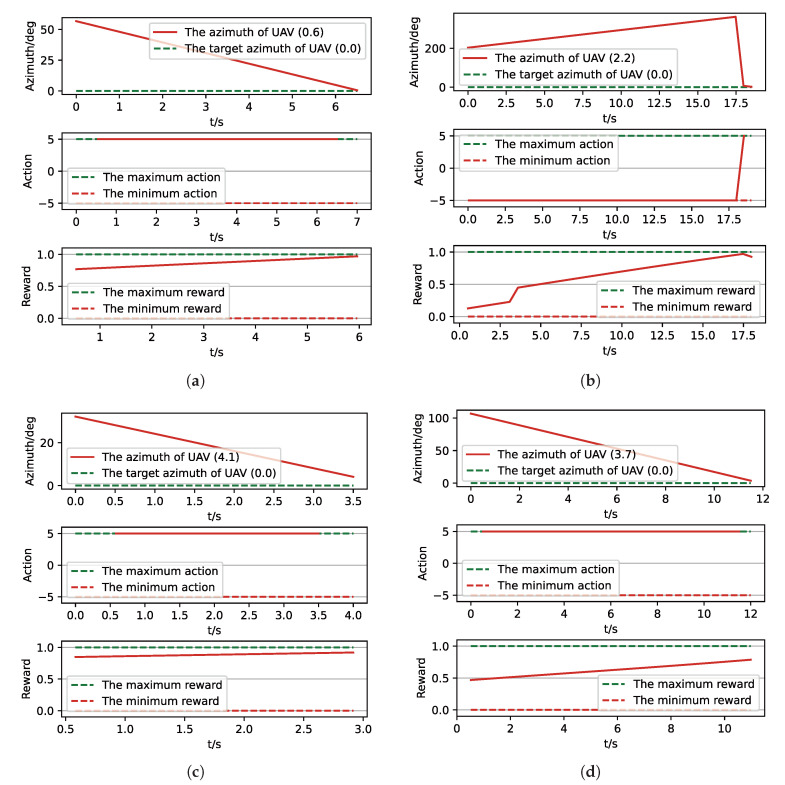
The parameters curve from MC experiments for the trained result of UER-DDPG. (**a**) The parameters curve of 1st experiment; (**b**) the parameters curve of 2nd experiment; (**c**) the parameters curve of 3rd experiment; (**d**) the parameters curve of 4th experiment.

**Figure 12:**
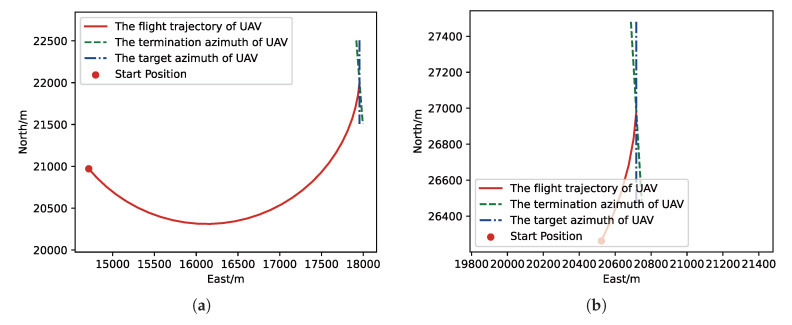

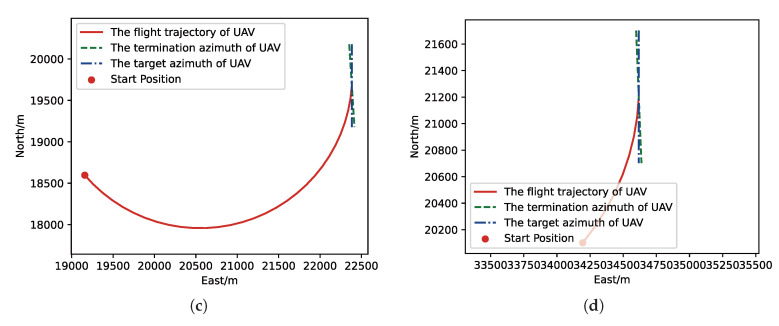
The flight trajectory from MC experiments for the trained result of PER-DDPG: (**a**) the flight trajectory of 1st experiment; (**b**) the flight trajectory of 2nd experiment; (**c**) the flight trajectory of 3rd experiment; (**d**) the flight trajectory of 4th experiment.

**Figure 13:**
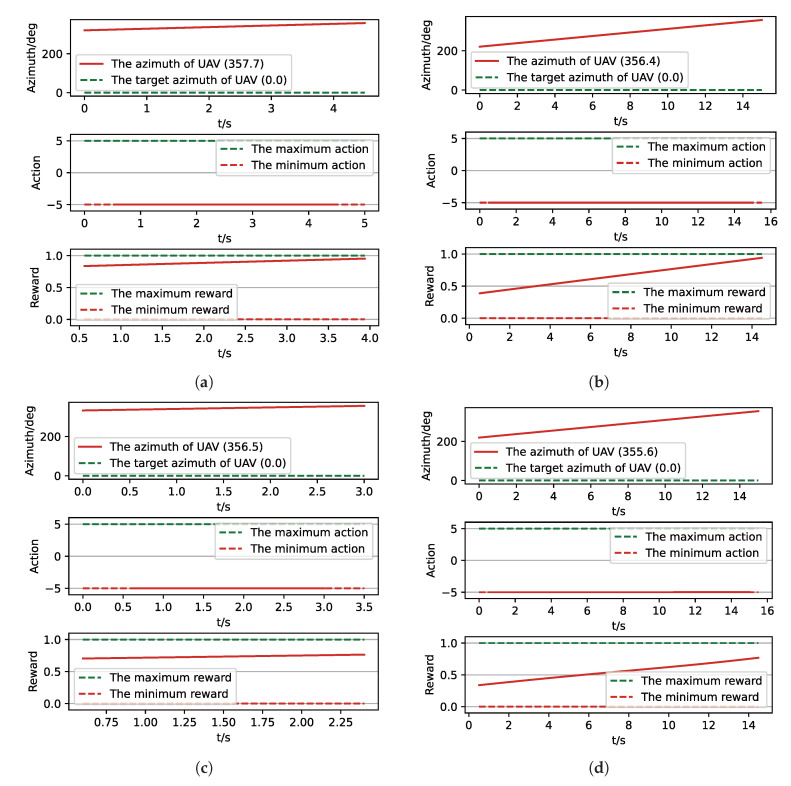
The parameters curve from MC experiments for the trained result of PER-DDPG: (**a**) The parameters curve of 1st experiment. (**b**) The parameters curve of 2nd experiment. (**c**) The parameters curve of 3rd experiment. (**d**) The parameters curve of 4th experiment.

After training, we run a group of Monte-Carlo experiments for trained results of UER-DDPG and PER-DDPG, and the number of MC experiments for each result is 1000. As shown in Table 5, the training result’s performance of PER-DDPG is better than UER-DDPG’s because the winning rate of PER-DDPG’s is more than about 3% than UER-DDPG’s. Meanwhile, we visualize some typical test results from MC experiments, and Figure 10, Figure 11, Figure 12 and Figure 13 is the flight trajectory of UAV and some parameters, including azimuth, reward, and action, over simulation step.

In Figure 10 and Figure 12 the red solid line represents the flight trajectory of UAV, and the red dashed line and the blue dash dot line indicate the termination azimuth of UAV and the required azimuth of LOS. In Figure 11 and Figure 13, the 1st row each figure is azimuth of UAV over simulation step, the 2nd row each figure is action of UAV over simulation step, and the 3rd row each figure is reward of UAV received over simulation step.

We can find that the algorithm based on PER-DDPG we proposed solves the turn round problem involved in airdrop task, and its performance is more than UER-DDPG. In summary, not only is the training process of algorithm based on PER-DDPG stabler than UER-DDPG’s, but also the trained result of algorithm based on PER-DDPG is much more effective than UER-DDPG’s.

### 3.3. The Simulation Results and Analysis of Guidance Problem

#### 3.3.1. The Parameters Setting of Algorithm

Based on content above, there are some parameters assignments of algorithm for guidance problem shown in Table 6. Moreover, according to the state space and action space of guidance problem, the structure of networks $Q(s,a;\theta^{Q})$ and $\mu (s;\theta^{\mu })$ is shown in Table 7 and Table 8, respectively.

#### 3.3.2. The Analysis of Simulation Results

**Figure 14:**
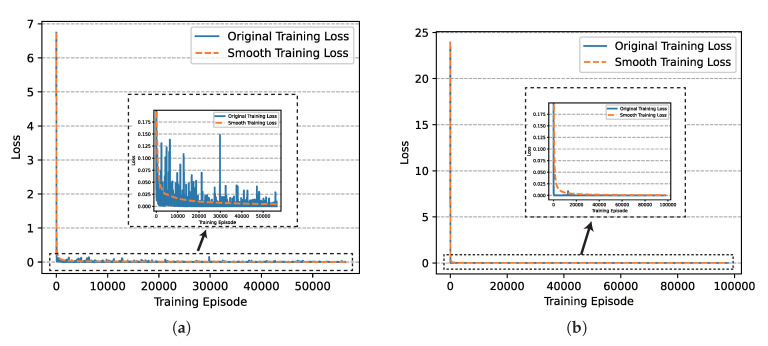
The comparison of critic networks’ training loss involved in UER-DDPG and PER-DDPG over training episode. (**a**) The training loss of critic network of UER-DDPG. (**b**) The training loss of critic network of PER-DDPG.

Similarly, we also analyzed the training loss, the winning rate, and the episode rewards generated during the training process of algorithms. In Figure 14, we could find that the convergence speed of PER-DDPG is more than UER-DDPG’s due to high utilization of experiences, and PER-DDPG becomes much stabler than UER-DDPG because the fluctuation of PER-DDPG is less than UER-DDPG.

**Figure 15:**
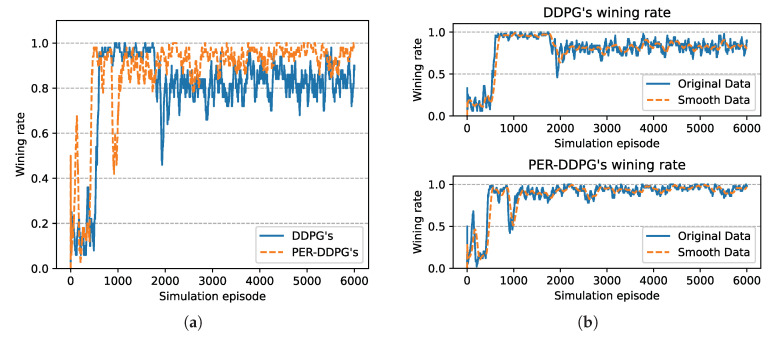
The winning rate of algorithms based on UER-DDPG and PER-DDPG over simulation episode. Winning rate means a rate of finishing mission successfully. (**a**) The comparison of winning rate based on UER-DDPG and PER-DDPG. (**b**) The figure in 1st row is the winning rate of algorithm based on UER-DDPG, and the figure in 2nd row is the winning rate of algorithm based on PER-DDPG.

**Figure 16:**
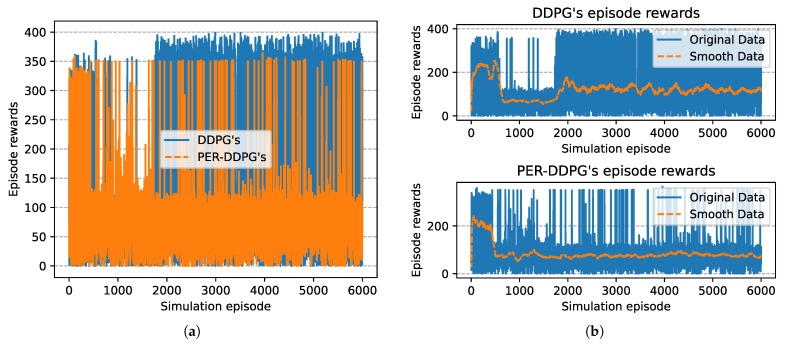
The episode rewards of algorithms based on UER-DDPG and PER-DDPG over simulation episode. The legends of figure are the same to Figure 9. (**a**) The comparison of episode rewards based on UER-DDPG and PER-DDPG. (**b**) The figure in 1st row is the episode rewards of algorithm based on UER-DDPG, and the figure in 2nd row is the episode rewards of algorithm based on PER-DDPG.

In Figure 15, the winning rate of algorithms based on different experience replay methods over simulation episode during the training process is shown. We can find that the winning rate curves of UER-DDPG and PER-DDPG are stable after some simulation episodes and maintain a high value. And the winning rate of PER-DDPG is much more than UER-DDPG after fluctuation. Meanwhile, the comparison of episode rewards could demonstrate that PER-DDPG is much steadier than UER-DDPG because the fluctuation of episode rewards of PER-DDPG is clearly less than UER-DDPG from Figure 16.

After training, we finished a set of Monte-Carlo experiments for trained results of UER-DDPG and PER-DDPG, and the number of it for each result is 1000. As shown in Table 9, the winning rate of PER-DDPG is more than approximately 3.5% than PER-DDPG, and we can think that the training result’s performance of PER-DDPG is better than UER-DDPG’s.

**Figure 17:**
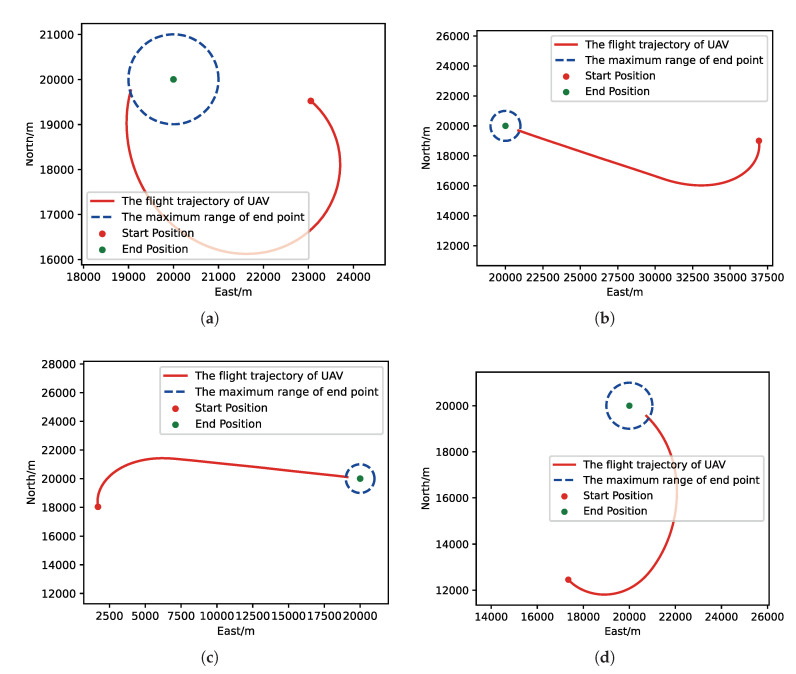
The flight trajectory from MC experiments for the trained result of UER-DDPG: (**a**) The flight trajectory of 1st experiment. (**b**) The flight trajectory of 2nd experiment. (**c**) The flight trajectory of 3rd experiment. (**d**) The flight trajectory of 4th experiment.

**Figure 18:**
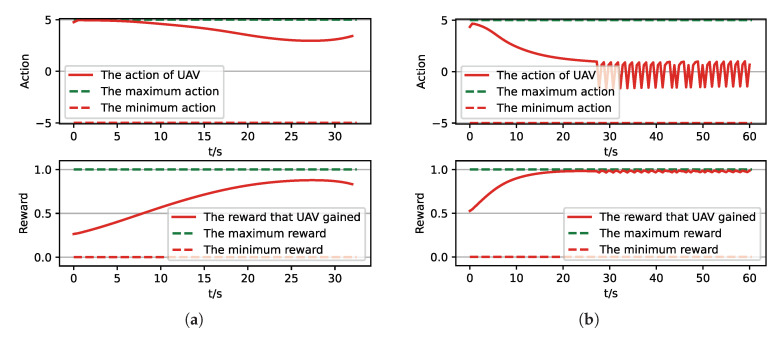

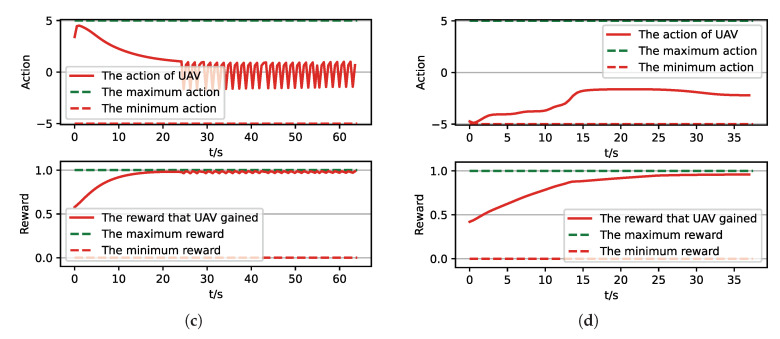
The parameters curve from MC experiments for the trained result of UER-DDPG. (**a**) The parameters curve of 1st experiment. (**b**) The parameters curve of 2nd experiment. (**c**) The parameters curve of 3rd experiment. (**d**) The parameters curve of 4th experiment.

**Figure 19:**
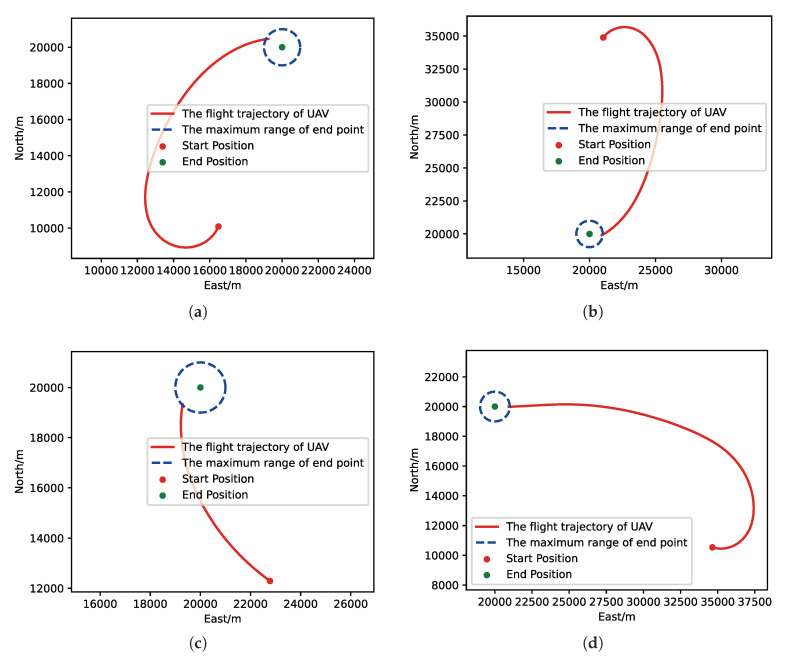
The flight trajectory from MC experiments for the trained result of PER-DDPG: (**a**) The flight trajectory of 1st experiment. (**b**) The flight trajectory of 2nd experiment. (**c**) The flight trajectory of 3rd experiment. (**d**) The flight trajectory of 4th experiment.

**Figure 20:**
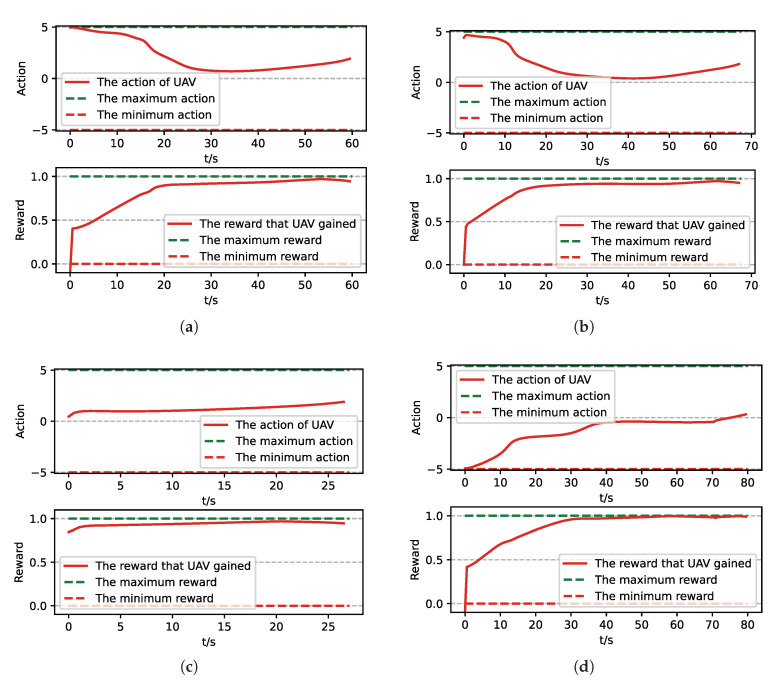
The parameters curve from MC experiments for the trained result of PER-DDPG: (**a**) The parameters curve of 1st experiment. (**b**) The parameters curve of 2nd experiment. (**c**) The parameters curve of 3rd experiment. (**d**) The parameters curve of 4th experiment.

Moreover, we visualize some typical test results from MC experiments in order to make our analysis more convincing. Figure 17, Figure 18, Figure 19 and Figure 20 is the flight trajectory of UAV and some parameters, including reward and action over simulation step. In Figure 17 and Figure 19, the red solid line represents the flight trajectory of UAV, and the blue dashed circle represents the maximum range of drop area. The red solid point and green solid point indicate start position and drop position. In Figure 18 and Figure 20, the top figure is the action of UAV over simulation step, the bottom figure is the reward of UAV received over simulation step.

According to results and analysis above, we could find that the algorithm based on PER-DDPG we proposed solves the guidance problem involved in airdrop task, and its performance is more than UER-DDPG. Similarly, not only is the training process of algorithm based on PER-DDPG stabler than UER-DDPG’s, but also the trained result of algorithm based on PER-DDPG is much more effective than UER-DDPG’s, while solving the guidance problem.

## 4. Conclusions

Aiming at the airdrop task, we refined and described two key issues, including turn round problem and guidance problem. Based on the definitions of problems, we designed the UAV maneuver decision-making model for airdrop task based on MDPs and constructed the state space, the action space, and the reward function based on PBRS. Then, we proposed the UAV maneuver decision-making algorithm for autonomous airdrop based on Deep Reinforcement Learning. Particularly, we used Prioritized Experience Replay to improve the availability of experiences during training process. Meanwhile, the results showed that the algorithm we proposed could be able to solve the turn round problem and guidance problem after training successfully. And the convergence of PER-DDPG is faster and stabler than UER-DDPG and the trained result performance of PER-DDPG is also better than UER-DDPG. In the future, we will investigate the solution of UAV autonomous flight when state is partially observed. And we will extend the algorithm we proposed to manipulate the real UAV to improve the autonomy of UAV, while performing special missions in the real world.

## Figures and Tables

**Table 1:** The details of data input into networks.

Parameter	Range	Meaning
$\delta_{\psi_{LOS}}$	$\left[0,2\pi \right)$	The relative azimuth between LOS and nose of UAV.
$N_{s}$	$\left[-N_{y}^{\max},N_{y}^{\max}\right]$	The steering overload of UAV.
$d_{LOS}$	$\left[0,D_{LOS}^{\max}\right]$	The distance between UAV and drop position.

**Table 2:** The parameters assignment of algorithm.

Parameter	Value	Meaning
*M*	1000	The number of simulation episodes.
*T*	300	The maximum of steps per episode.
*K*	32	The training episode of algorithm.
*k*	32	The size of training batch.
$D_{size}$	5000	The size of experiences memory D.
α	0.5	The availability exponent of PER.
$\beta_{0}$	0.4	The initial exponent of IS.
$\beta_{inc}$	0.0001	The increment of exponent of IS.

**Table 3:** The structure of critic network $Q(s,a;\theta^{Q})$.

Layers	Layer Structure	
	Units	Activation Function
Input layer	3	-
Hidden layer 1	32	ReLU
Hidden layer 2	64	ReLU
Output layer	1	-

**Table 4:** The structure of actor network $\mu (s;\theta^{\mu })$.

Layers	Layer Structure	
	Units	Activation Function
Input layer	2	-
Hidden layer 1	32	tanh
Hidden layer 2	64	tanh
Output layer	1	tanh

**Table 5:** The analysis of MC experiments for the trained results of UER-DDPG and PER-DDPG.

Sampling Method	Winning Rate
UER	92.9%
PER	95.3%

**Table 6:** The parameters assignment of algorithm.

Parameter	Value	Meaning
*M*	6000	The number of simulation episodes.
*T*	1000	The maximum of steps per episode.
*K*	64	The training episode of algorithm.
*k*	64	The size of training batch.
$D_{size}$	10,000	The size of experiences memory *D*.
α	0.5	The availability exponent of PER.
$\beta_{0}$	0.4	The initial exponent of IS.
$\beta_{inc}$	0.0001	The increment of exponent of IS.

**Table 7:** The structure of critic network $Q(s,a;\theta^{Q})$.

Layers	Layer Structure	
	Units	Activation Function
Input layer	3	-
Hidden layer 1	32	ReLU
Hidden layer 2	64	ReLU
Hidden layer 3	64	ReLU
Hidden layer 4	64	ReLU
Output layer	1	-

**Table 8:** The structure of actor network $\mu (s;\theta^{\mu })$.

Layers	Layer Structure	
	Units	Activation Function
Input layer	2	-
Hidden layer 1	32	tanh
Hidden layer 2	64	tanh
Hidden layer 3	64	tanh
Output layer	1	tanh

**Table 9:** The analysis of Monte-Carlo (MC) experiments for the trained results of UER-DDPG and PER-DDPG.

Sampling Method	Winning Rate
UER	93.2%
PER	96.8%
